# Supplementary material for: TGFß/activin-dependent activation of Torso controls the timing of the metamorphic transition in the red flour beetle Tribolium castaneum
Source: PLoS Genet. 2023 Nov 27;19(11):e1010897. doi: 10.1371/journal.pgen.1010897 (PMC10703416; doi:10.1371/journal.pgen.1010897)
Supplement: Attachment — Submitted filename: ResponseReferees.docx [file pgen.1010897.s002.docx]

**Dr. Xavier Franch-Marro**

Senior Scientist of CSIC

Institute of Evolutionary Biology (IBE)

CSIC- Pompeu Fabra University

Barcelona Biomedical Research Park (PRBB)

Barcelona, November 3^th^, 2023

Dear Prof. Riddiford,

We are very pleased by your letter reporting the positive evaluation from the two reviewers and the possibility to response to their comments.

We read carefully all the comments and suggestions from all reviewers and we would like to thank them for the constructive task conducted. We have addressed all points raised by the three Reviewers and incorporated into the manuscript, all comments and suggestions. Specially we confirmed the different tissue specific expression of the two Tc-Torso ligands, Tc-Ptth and Tc-Trk. In addition, we confirmed the additional role of TGFβ/Activin controlling ecdysone biosynthesis by simultaneously knockdown *Tc-jhamt* in *Tc-myo* and *Tc-babo* depleted larvae as suggested by the referees. All new data generated has been incorporated in two new additional figures (Fig 3 and 7), two new panels in Figure 5, and in the final version of the manuscript. Therefore, we think that the reviewed version of the manuscript satisfied the requirements of the journal.

Please, find enclosed the detailed answers to all the points raised by the reviewers. We believe that as requested, we addressed all the points raised satisfactorily and we are looking forward to receive your definitive positive answer.

Thank you very much for your consideration,

Sincerely,

**Dr. Xavier Franch-Marro and Dr.David Martín**

Senior Scientists of CSIC

Institute of Evolutionary Biology (IBE)

CSIC- Pompeu Fabra University

Barcelona Biomedical Research Park (PRBB)

**Reviewer #1: In the current manuscript, Chafino and others describe a signaling network that controls the timing of metamorphosis in the red flour beetle Tribolium castaneum. The authors present compelling evidence suggesting that the metamorphosis timing is differentially regulated by two ligands of the receptor tyrosine kinase named Torso, whose expression is in turn induced by the decline of juvenile hormone (JH). They also demonstrate that JH production in the last instar Tribolium is suppressed by a TGF-beta ligand named Myoglianin. By suppressing JH signaling and thereby promoting Torso expression, Myoglianin functions as a critical initiator of metamorphosis. Their model is consistent with the fact that Myoglianin initiates metamorphosis in many insect species, and it provides an important framework for understanding this critical process of insect development.**

**Overall, experiments are carefully designed and thoroughly conducted, making their proposed model convincing. Although there is some remaining uncertainty regarding specific functions of some genes such as Tc-torso, it requires development of new genetic tools for them to fully address those remaining issues. Having said that, I would like to propose two additional experiments that are still doable with available molecular tools, which I believe will significantly strengthen their argument. They are described below as two major comments:**

We are very pleased that the reviewer 1 found the work interesting and relevant to the field.

**Major comments:**

**1) Although their claim that Tc-Torso has two ligands in the last instar Tribolium is exciting, currently their claim is not convincing enough, as they do not provide any data that can explain how two ligands of a single receptor can have different functions. As the authors propose differential functions of Torso signaling in two different tissues (the PG and fat body), it would be interesting to check tissue specific expression patterns of Torso, as well as its two ligands, PTTH and Trunk. I understand that the PG is not identified in Tribolium yet, but the authors can still check the expression levels of these genes in the CNS and fat body, as well as some other tissues. I guess PTTH is specifically expressed in the CNS, but how about Trunk? Is it expressed in peripheral tissues, such as the fat body? Is Tc-torso really expressed in the fat body? This is a simple and doable experiment, whose outcome will be useful to interpret some of the presented data.**

This is an intriguing observation, and we concur with the referee's suggestion that acquiring such data could provide substantial support for distinguishing the distinct roles of Tc-Trunk and Tc-Ptth in *Tribolium*. As the referee suggested, we conducted a series of dissections involving various tissues in L7 specimens to quantify the expression levels of both ligands. Given the unlocalized nature of the PG in *Tribolium*, we opted to dissect the entire head and the first thoracic segment to ensure that we captured this elusive organ. Additionally, we performed dissections on muscle, fat body and gut. Interestingly, we observed distinct tissue-specific expression patterns for these two ligands. While *Tc-Ptth* mRNA is predominantly enriched in the head, *Tc-trk* was exclusively detected in the gut and fat body, two of the primary nutrient-sensing tissues. These findings support the possibility of different ligand-specific activation of Tc-Torso in *Tribolium*. We have incorporated this information into the manuscript and have also included it in a new Figure 3.

**2) Currently, it is unclear whether (and to what extent) the low ecdysone signaling in babo-RNAi and myo-RNAi L7 larvae can be explained by the high JH titer in these animals on the PG. Although suppression of torso expression by JH alone cannot explain the strong arrest phenotype of these RNAi larvae, it is possible that JH can suppress ecdysone production in the PG in multiple ways. It is therefore very interesting to try double knockdown of babo and jhamt, as well as myo and jhamt in L7 larvae. The results of this experiment would tell us whether myo-babo signaling directly promotes ecdysone production in the PG, or the effect is rather indirect through its suppression of the JH titer.**

Once again, the referee has raised a highly intriguing point that could significantly contribute to our understanding of the TGF-ß/Activin mode of action in the PG. Following the reviewer's recommendation, we conducted simultaneous injections of *dsTc-myo* and *dsTc-jhamt* as well as *dsTc-babo* and *dsTc-jhamt* into late L6 larvae. As expected, inhibition of JH biosynthesis in a TGF-ß/Activin signalling pathway inactivation background, proved ineffective in rescuing the arrested development induced by the absence of either *Tc-myo* or *Tc-babo*. This result aligns with the observation that the application of the JH analog Methoprene during the last larval instar of *Tribolium*, while blocking metamorphosis, does not impair larval molting (our data and Ko C, Chaieb A. et al. PLoS Genetics, 2014), suggesting that, under these conditions, the production of ecdysone is not abolished. In summary, these results confirm that TGF-ß/Activin signalling directly promotes ecdysone production and very likely contributes to PG integrity. We have incorporated this information into the manuscript and have also included it in a new Figure (Figure 7).

**Minor comments:**

**3) p. 5, line 75: “increased” should be “increase.”**

We fixed this mistake.

**4) p. 5, line 77: “Myostatin” should be “Myoglianin.”**

We changed Myostatin to Myoglianin as suggested.

**5) p. 6, lines 122-123, “(Tc-Ptth RNAi animals) induced a pupariation delay of 4 days, compared to the 7 days observed in Tc-torso RNAi larvae”: I am not sure if this statement is accurate based on the data shown in Figure 2A. It appears that the difference of their median values is less than two days. Also, please label the Y axis of Figure 2A with tick marks, so we can read the data more accurately.**

We apologize for the mistake. As the referee pointed out, the difference in pupation delay between *Tc-torso* and *Tc-Ptth* is less than 2 days. This error resulted from incorrectly introducing the data into the manuscript by using the average of the developmental delay instead of the median, as indicated in the plot. We have rectified this mistake in the manuscript and incorporated ticks in panel A of Fig 2, as suggested.

**6) p. 7, line 140: “puparium” should be either “pupation” or “pupariation.” Also, it seems that these two words are mixed throughout the manuscript (e.g. the Y axis of Figure 2A is labeled “pupariation”, but the authors say that larvae “pupated,” for example on p. 6, line 113. This wording needs to be standardized.**

We meticulously reviewed the manuscript and employed "pupation" consistently throughout the text.

**7) p. 8, line 186: The word “Torso” is missing.**

We fixed this mistake.

**8) p. 10, line 231: “Figure 5E and E” should be “Figure 5E and F.”**

We changed the labelling as suggested.

**9) p. 13, lines 322-324, the sentence “Thus, … jhamt [31, 32, 42]”: I guess “inactivation of TGFbeta/Activin signaling” here should be just “TGFbeta/Activin signaling.” Otherwise, it is the opposite of what they have shown in their current study. Also, “directly downregulation of” should be “directly downregulating.”**

We modified the sentence as suggested.

**Reviewer #2: The manuscript by Chafino et al., investigates the role of the PTTH, Trunk and Myo signaling molecules in regulating entry of T castaneum larva into metamorphosis. PTTH and Trk are the two know ligands for the receptor tyrosine kinase receptor Torso, while Myo is one of the known TGF beta /Activin-like ligands that signal through the type I receptor Babo. Both Torso and Babo signaling has been implicated previously in regulating the metamorphic transition in several holometabolous insects, but details of molecular interplay between these signaling pathways has remained elusive. In this manuscript, the authors use gene expression levels and RNAi knockdown methods to tease apart the interactions between these two signaling pathways that control initiation of metamorphosis in Tribolium. They two major new discoveries. The first is that, unlike Drosophila where Trk and PTTH have very stage specific and non-overlapping functions during embryogenesis and metamorphosis respectively, In Tribolium Trk also activates Torso during the larval phase. Specifically, it appears to specifically regulate larval growth during the last larval stage while PTTH is responsible for triggering metamorphosis. This is a very intriguing discovery and the interpretation with regards to evo/dev mechanisms in the discussion is quite enlightening. The second and perhaps more important new mechanistic finding is that way in which the Myo/Babo pathway intersects with the PTTH/Trk pathway. The authors convincingly show that Myo /Babo signaling is responsible for shutting down JH synthesis at the end of the larval phase. This in turn stimulates high level expression of Torso so that, in response to PTTH, ecdysone biosynthesis is enhanced. This finding validates - at the molecular mechanism level- the long held textbook view that it is a combination of low JH and high E the stimulates the metamorphic transition in holometabolous insects. Overall, I find the experiments to be well done and the interpretations convincing.**

**It is too bad that the genetics are not far enough along in Tribolium to look at the interaction in more detail such as what is the tissue source of the Myo (muscle??) and is Babo expressed and required in the CA for JH downregulation. However, even without these additional details, I think the paper contributes a major advance to our understanding of the signaling cascades that regulate production of the two key hormones that trigger metamorphosis in holometabolous insects.**

As before, we thank Prof O’Connor for his suggestions and comments and to find our work of interest**.**

**Some minor comments**

**Since basal levels of Ecdysone production are required for imaginal disc growth in Drosophila especially during the last instar, is it possible that Tc -trk exerts its effects on the L7 growth via the basal level of ecdysone production and not via an effect of Trk signaling through Torso in another tissue (fatbody) as suggested in the discussion? What is the phenotype of knocking down an E biosynthetic enzyme in the final instar at different times. If one knocks down early is the body size (growth rate) affected differentially compared to knockdown in the last couple of days of L7?**

The notion that Tc-Trk activates Tc-Torso in the PG to ensure an adequate ecdysone supply for normal growth is certainly a plausible hypothesis. In this context, systemic depletion of Ecr (Palli et al. MolCellEndocrinol, 2008) or phm (as observed in our data) in the last larval stage results in smaller larvae that subsequently die during the quiescent stage. However, the distinct expression pattern of *Tc-trk* in the gut and fat body also lends support to the possibility of Tc-Torso being activated specifically in those tissues. Unfortunately, addressing these questions is currently unfeasible, as specific tools for depleting *Tc-torso* in these tissues are not yet available. We discuss this interesting possibility in the discussion.

**Is it possible to inject Torso mRNA into starved larva to see if it can bypass the block in attaining the TS threshold?**

This is indeed an intriguing suggestion. However, it is currently unfeasible to perform such a technique in Tribolium.

**If the function of Babo/Myo signaling is primarily to downregulate JH signaling in the last instar, one might predict that double knockdown of babo and Tc-Kr-h1 might allow normal molting. Have the authors tried his experiment?**

Once again, this is a very interesting point that we have addressed in the revised manuscript. Please also refer to our response to referee 1.

**Minor comments:**

**Page 6 line 114, should read Dm-PTTH and Dm-Torso not Tc-Ptth and Tc-torso.**

We changed that.

**Page 6 115-116 the sentence starting “These results suggest …. Is awkward using the parenthesis. It would read better to express as two separate thoughts in two sentences.**

We changed that.

**Page 8 line 186 TGFbeta/Activin signaling pathway regulates (insert Torso) expression…**

We added Tc-Torso in the sentence as suggested.

**Page 13 line 340 …knocked down…**

We fixed that.

**Reviewer #3: In this study, Chafino et al explore the molecular regulation of the threshold size. They convincingly demonstrate that Torso expression is upregulated upon threshold size attainment and that it is only required during the final instar. Furthermore, they show that Torso mediates the effect of PTTH on Halloween gene expression and Trk on final instar growth. Finally, they demonstrate that the Torso expression is regulated by TGF-b/Activin signaling. The identification of Torso as a final instar-specific marker for the attainment of threshold size is novel and interesting. The study is well designed, and the data are clearly presented. I do have several issues that I would like the authors will address.**

We would like to thank the referee for all the task conducted and to find our work of interest.

**Major comments**

**- The referenced figures do not seem to point to the correct figures in some places. For example, in line 115, Fig 2C-E are listed as showing the smaller body size of Torso RNAi pupae. This should just be Figs 2D and E. In addition, in line 121, Fig. 2C is listed as showing the expression of PTTH; however, in the figure, PTTH is actually shown in Fig 2B. In line 173, I believe Fig. 3C should be cited instead of Fig. 3B. Please double check to make sure the referenced figures are accurate.**

We apologize for these oversights. We have thoroughly reviewed the manuscript and ensured that the figures are cited correctly.

**- The expression profile of trk is interesting as it exhibits a dramatic drop on day 4. Is there a significance here? For example, does it correspond to prepupal entry and cessation of feeding (similar to how insulin might respond to nutritional inputs)? If possible, it would be nice to have prepupal stage indicated in the expression profiles.**

This is a highly intriguing observation. The downregulation of *Tc-trk* aligns with the entry into the quiescent stage, further supporting the concept that *Tc-trk* responds to nutritional cues to promote growth. We have incorporated this point into the discussion section and have also explicitly indicated the quiescent/prepupal stage in all expression profiles, as recommended.

**- Line 176-179: When jhamt3 and torso are simultaneously knocked down, the developmental delay increases from 5 days to 7 days. This is modest compared to the developmental delay seen in the final instar when torso alone is depleted (5->10 days). Given this, it might strengthen your arguments if you demonstrated that jhamt3 knockdown during L5 leads to precocious upregulation of torso in L6. I think such an experiment would be useful in demonstrating that torso is always upregulated in the instar prior to pupation. I don’t think this is absolutely necessary for publication, however, so I will leave this at the authors’ discretion.**

The difference in developmental delay observed between the depletion of *Tc-torso* during the last larval stage and the simultaneous knockdown of *Tc-jhamt* and *Tc-torso* can be attributed to the challenges associated with injecting two dsRNAs into very early larval stages. In such cases, the quantity of dsRNA injected is lower, potentially rendering the treatment less effective.

Regarding the measurement of *Tc-torso* in *dsTc-jhamt* L4-injected larvae, while it is an intriguing idea, it presents significant challenges. The substantial variability in developmental delay under these conditions makes it difficult to collect a sufficient number of larvae at the precise developmental stage for accurate measurements.

**- Line 300-306: The role of JH in Drosophila larvae is minimal (see Riddiford and Ashburner 1991, Gen. Comp. Endocrin. 82:172-183.). Given this, I would be cautious about making comparisons with Drosophila when it pertains to JH’s regulation of metamorphic onset. In many other insects, there is a “critical weight” after the “threshold weight” that is associated with the complete removal of JH from the hemolymph and hence entry into the prepupal stage. In these insects, PTTH can only be secreted after the critical weight has been reached (Nijhout and Williams, 1974, J Exp Biol 61: 493–501). I think the regulation of metamorphic transition in Tribolium is likely to be more similar to lepidopterans than it would be to Drosophila. I do agree that JH does appear to suppress Torso and the BASAL ecdysteroid biosynthesis in the final instar, and this effect is likely similar to what is observed in Bombyx (i.e. lines 307-311).**

We concur with the referee that Drosophila exhibits a unique pattern of development and that the significance of JH is comparatively less pronounced than in other insect species. However, it is worth noting that recent reports have highlighted the role of JH in suppressing the expression of Halloween genes in the PG (Liu S et al. PNAS, 2018 and Zhang T. et al. PNAS, 2018). Therefore, we would like to emphasize that the function of JH signaling in repressing ecdysone biosynthesis is a conserved phenomenon in insects, including *Drosophila*.

**- The above comment brings up another issue, which is that in lepidopterans (and in Drosophila), the activation of Halloween genes is associated with the attainment of the minimum viable weight, which is reached relatively early after insect start feeding in the final instar and is attained after the threshold size is reached (see for example, Xu et al 2020. Insect Biochem Mol Biol 119, 103335). I therefore wonder if torso is upregulated at the threshold size or at the minimum viable weight? I just want to make sure the equivalent stages are named appropriately to avoid confusion in the future.**

The term "Minimal Viable Weight" (MVW) signifies the minimum weight necessary to initiate pupation and successfully undergo metamorphosis. In species like *Tribolium* and *Bombyx*, this MVW is typically reached during early larval stages. Specifically, in *Tribolium*, MVW is attained at the fifth larval instar (L5), a stage when the expression of E93 can be induced. However, under normal conditions, metamorphosis occurs two or three larval stages later due to the repressive effect of JH signaling on E93 expression. Therefore, premature suppression of juvenile hormone (JH) biosynthesis leads to the precocious expression of E93, subsequently inducing metamorphosis after L5 (Chafino et al., Cell Reports 2019). In contrast, the term "Threshold Size" (TS) refers to the weight required to trigger metamorphosis once MVW is achieved. In *Tribolium*, TS is typically reached within the first 24 h after the molt into the last larval stage (L7), which results in the expression of E93 and the concurrent repression of Kr-h1. Finally, "Critical Weight" (CW) denotes the weight necessary to induce the subsequent molt, both in the transition from larva to larva and from larva to pupa. Notably, in Drosophila, MVW and TS are reached at the same developmental time point, early in the third larval instar, and are sometimes incorrectly referred to as CW. Interestingly, in both *Tribolium* and *Drosophila*, and likely in Bombyx as well, the expression of Torso is activated after achieving the TS.

**- Line 351-367: Here the authors explain the inability of TcBabo RNAi larvae to molt by proposing that the PGs might degenerate through autophagy. Specifically, they discuss the effect of inactivation of TGF-beta signaling on adult Drosophila cardiac muscles and suggest that TcMyo may be needed for PG survival. While possible, considering the fact that the knockdown of Myo in Blattella leads to hyperproliferation of PG cells and an increase in the PG size (Kamsoi and Belles, 2019, FASEB J. 33, 3659–3669), I would tend to favor the idea that the Halloween gene expression remains so low that ecdysteroid levels never reaches a high enough level to trigger a molt. Consequently, I would suggest discussing these other possibilities and changing the subheading of this section.**

We concur with the referee's perspective that blocking TGFB/activin signaling should not significantly impact cell viability and could, in fact, induce hyperproliferation, similar to what occurs in Blattella. However, we must acknowledge our limitations in addressing this question adequately due to the fact that the Tribolium PG remains unidentified. Nonetheless, the considerable developmental delay of more than 60 days resulting from the injection of dsTc-myo makes it improbable that the inactivation of TGFB/activin induces PG overproliferation for several reasons. Firstly, in contrast to *Tribolium*, the depletion of *Bg-myo* in *Blattella* allows for multiple supernumerary moltings without causing a significant developmental delay. Secondly, the effects of silencing *Bg-babo* were not analyzed in *Blattella*, thus not excluding a possible stronger effect. Consequently, we lean towards the hypothesis that TGFB/activin signaling is essential for PG viability. However, we have included this alternative possibility in the discussion section of the manuscript and revised the subheading of the section to pose it as a question, emphasizing that it remains a potential explanation.

**- Fig. 4 legend states that t-tests were used to compare the mean expression changes. Please specify what the two treatment groups are since there are more than two different treatment groups shown and t-tests should only be used for comparisons between two treatment groups.**

We have added this information in the Figure legends

**Minor comments**

**Middle of the abstract: “Our data reveals” should be “Our data reveal”**

We changed that.

**Line 10: “Holomeabolous:” should be “Holometabolous”**

We fixed that.

**Line 77” The authors use “Myostatin” as the name of the ligand. Although TcMyo is a homolog of Myostatin, it is also a homolog of GDF11. Later on, the authors use the term Myoglianin (line 193). I would suggest that the authors stick with Myoglianin throughout the manuscript.**

We used Myoglianin throughout the manuscript as suggested.

**Line 117: “might not only depends” should be “might not only depend”**

We rephrased the sentence.

**Line 120: Tribolium should be italicized**

We fixed that.

**Line 181: “trough” should be “through”**

We changed that.

**Line 219: “when we stop analyzing the knockdown animals” – change to ““when we stopped analyzing the knockdown animals”**

We changed that.

**Line 223: “On the other hand” should follow “on the one hand”. Perhaps use “In contrast”?**

We changed that.

**Line 231: “Fig. 5 E and F” instead of “5E and E”?**

We fixed that.

**Line 227-228: I would suggest including Blattella to this list as well (Kamsoi and Belles 2019).**

We added the refrence as recommended.

**Line 232: “Collectively”**

We changed that.

**Line 243: “Trk promotes organ growth”. Can you say this with such certainty? Depletion of Trk leads to smaller body size, so this may be due to differences in organ growth, but one cannot necessarily rule out lowered feeding rates, for example.**

We rephrased the sentence.

**Line 270: “this data is consistent” should be “these data are consistent”**

We changed that.

**Line 323: “blocks JH production by directly downregulation of…”: Change to “blocks JH production by directly downregulating…”**

We changed that.

**Line 332: “rise” instead of “raise”**

We fixed that.

**Line 339: “inter-molting period” should be “intermolt period”**

We changed that.

**Line 374: Italicize Tribolium**

We changed that.

**Line 414: Please give the exact amount of dsRNA injected.**

We added this information in materials and methods.

**Line 429, 601: Italicize Tribolium**

We have done that

**Line 603, 619: Italicize Tc-Rpl32**

We have done that

**Line 617: Instead of just (B), it should probably be (B, C)**

We have changed that.

**Fig 3 legend: The legend for 3A describes t-tests and asterisks. However, it looks like an ANOVA was used instead and letters are provided. Please remove line 634.**

The referee is correct, and we apologize for the mistake. We have removed the sentence.

**Line 670: Instead of just (E), it should probably be (E, F)**

We have relocated these panels to Figure 7 with proper labeling.

**Fig. 1, 2 legends: Please define pd0 in the expression profile.**

We added this information in Figure 1 and 2 as suggested.
